# Supplementary material for: HIVprotI: an integrated web based platform for prediction and design of HIV proteins inhibitors
Source: J Cheminform. 2018 Mar 9;10:12. doi: 10.1186/s13321-018-0266-y (PMC5845081; doi:10.1186/s13321-018-0266-y)
Supplement: Supplementary file 2 — Additional file 2. Source code of HIVProtI web server. [file 13321_2018_266_MOESM2_ESM.zip › HIVprotI_Source-code/hivprotI/weka-3-6-6/doc/weka/classifiers/bayes/net/search/ci/ICSSearchAlgorithm.html]

ICSSearchAlgorithm


---


|  |  |  |
| --- | --- | --- |
| **Overview** \| **Package** \| **Class** \| **Tree** \| **Deprecated** \| **Index** \| **Help** \| **Weka's home** | |  |
| **PREV CLASS**   NEXT CLASS | **FRAMES**    **NO FRAMES**     **All Classes** |  |
| SUMMARY: NESTED \| FIELD \| CONSTR \| METHOD | DETAIL: FIELD \| CONSTR \| METHOD |  |


---


## weka.classifiers.bayes.net.search.ci Class ICSSearchAlgorithm

```
java.lang.Object
  weka.classifiers.bayes.net.search.SearchAlgorithm
      weka.classifiers.bayes.net.search.local.LocalScoreSearchAlgorithm
          weka.classifiers.bayes.net.search.ci.CISearchAlgorithm
              weka.classifiers.bayes.net.search.ci.ICSSearchAlgorithm
```

**All Implemented Interfaces:**: java.io.Serializable, OptionHandler, RevisionHandler

---

``` public class ICSSearchAlgorithm extends CISearchAlgorithm ```

This Bayes Network learning algorithm uses conditional independence tests to find a skeleton, finds V-nodes and applies a set of rules to find the directions of the remaining arrows.


Valid options are:

```
 -cardinality <num>
  When determining whether an edge exists a search is performed 
  for a set Z that separates the nodes. MaxCardinality determines 
  the maximum size of the set Z. This greatly influences the 
  length of the search. (default 2)
```

```
 -mbc
  Applies a Markov Blanket correction to the network structure, 
  after a network structure is learned. This ensures that all 
  nodes in the network are part of the Markov blanket of the 
  classifier node.
```

```
 -S [BAYES|MDL|ENTROPY|AIC|CROSS_CLASSIC|CROSS_BAYES]
  Score type (BAYES, BDeu, MDL, ENTROPY and AIC)
```

**Version:**
:   $Revision: 1.8 $

**Author:**
:   Remco Bouckaert

**See Also:**: Serialized Form

---

**Field Summary**

**Fields inherited from class weka.classifiers.bayes.net.search.local.LocalScoreSearchAlgorithm**

`TAGS_SCORE_TYPE`


**Constructor Summary**

`ICSSearchAlgorithm()`


| **Method Summary** | |
| --- | --- |
| `int` | `getMaxCardinality()`             returns the max cardinality |
| `java.lang.String[]` | `getOptions()`             Gets the current settings of the Classifier. |
| `java.lang.String` | `getRevision()`             Returns the revision string. |
| `java.lang.String` | `globalInfo()`             This will return a string describing the search algorithm. |
| `java.util.Enumeration` | `listOptions()`             Returns an enumeration describing the available options. |
| `static void` | `main(java.lang.String[] argv)`             for testing the class |
| `java.lang.String` | `maxCardinalityTipText()` |
| `void` | `setMaxCardinality(int nMaxCardinality)`             sets the cardinality |
| `void` | `setOptions(java.lang.String[] options)`             Parses a given list of options. |

**Methods inherited from class weka.classifiers.bayes.net.search.local.LocalScoreSearchAlgorithm**

`buildStructure, calcNodeScore, calcScoreWithExtraParent, calcScoreWithMissingParent, getMarkovBlanketClassifier, getScoreType, logScore, markovBlanketClassifierTipText, scoreTypeTipText, setMarkovBlanketClassifier, setScoreType`

**Methods inherited from class weka.classifiers.bayes.net.search.SearchAlgorithm**

`initAsNaiveBayesTipText, maxNrOfParentsTipText, toString`

**Methods inherited from class java.lang.Object**

`equals, getClass, hashCode, notify, notifyAll, wait, wait, wait`

**Constructor Detail**

### ICSSearchAlgorithm

```
public ICSSearchAlgorithm()
```


**Method Detail**

### setMaxCardinality

```
public void setMaxCardinality(int nMaxCardinality)
```

:   sets the cardinality

    :   **Parameters:**: `nMaxCardinality` - the max cardinality

---


### getMaxCardinality

```
public int getMaxCardinality()
```

:   returns the max cardinality

    :   **Returns:**: the max cardinality

---


### listOptions

```
public java.util.Enumeration listOptions()
```

:   Returns an enumeration describing the available options.

    :   **Specified by:**: `listOptions` in interface `OptionHandler` **Overrides:**: `listOptions` in class `LocalScoreSearchAlgorithm`
    :   **Returns:**: an enumeration of all the available options.

---


### setOptions

```
public void setOptions(java.lang.String[] options)
                throws java.lang.Exception
```

:   Parses a given list of options.


    Valid options are:

    ```
     -cardinality <num>
      When determining whether an edge exists a search is performed 
      for a set Z that separates the nodes. MaxCardinality determines 
      the maximum size of the set Z. This greatly influences the 
      length of the search. (default 2)
    ```

    ```
     -mbc
      Applies a Markov Blanket correction to the network structure, 
      after a network structure is learned. This ensures that all 
      nodes in the network are part of the Markov blanket of the 
      classifier node.
    ```

    ```
     -S [BAYES|MDL|ENTROPY|AIC|CROSS_CLASSIC|CROSS_BAYES]
      Score type (BAYES, BDeu, MDL, ENTROPY and AIC)
    ```

    :   **Specified by:**: `setOptions` in interface `OptionHandler` **Overrides:**: `setOptions` in class `LocalScoreSearchAlgorithm`
    :   **Parameters:**: `options` - the list of options as an array of strings **Throws:**: `java.lang.Exception` - if an option is not supported

---


### getOptions

```
public java.lang.String[] getOptions()
```

:   Gets the current settings of the Classifier.

    :   **Specified by:**: `getOptions` in interface `OptionHandler` **Overrides:**: `getOptions` in class `LocalScoreSearchAlgorithm`
    :   **Returns:**: an array of strings suitable for passing to setOptions

---


### maxCardinalityTipText

```
public java.lang.String maxCardinalityTipText()
```

:   **Returns:**: a string to describe the MaxCardinality option.

---


### globalInfo

```
public java.lang.String globalInfo()
```

:   This will return a string describing the search algorithm.

    :   **Overrides:**: `globalInfo` in class `CISearchAlgorithm`
    :   **Returns:**: The string.

---


### getRevision

```
public java.lang.String getRevision()
```

:   Returns the revision string.

    :   **Specified by:**: `getRevision` in interface `RevisionHandler` **Overrides:**: `getRevision` in class `CISearchAlgorithm`
    :   **Returns:**: the revision

---


### main

```
public static void main(java.lang.String[] argv)
```

:   for testing the class

    :   **Parameters:**: `argv` - the commandline parameters


---


|  |  |  |
| --- | --- | --- |
| **Overview** \| **Package** \| **Class** \| **Tree** \| **Deprecated** \| **Index** \| **Help** \| **Weka's home** | |  |
| **PREV CLASS**   NEXT CLASS | **FRAMES**    **NO FRAMES**     **All Classes** |  |
| SUMMARY: NESTED \| FIELD \| CONSTR \| METHOD | DETAIL: FIELD \| CONSTR \| METHOD |  |


---
